# Supplementary material for: Patterns of Intron Gain and Loss in Fungi
Source: PLoS Biol. 2004 Nov 30;2(12):e422. doi: 10.1371/journal.pbio.0020422 (PMC532390; doi:10.1371/journal.pbio.0020422)
Supplement: Table S1 — Also available at http://genes.mit.edu/NielsenEtAl/. (4.3 MB ZIP). [file pbio.0020422.st001.zip › NielsenEtAl/html/873.html]

AN3033.1.NCU02232.1.MG00881.1.FG06339.1


```
 CLUSTAL W (1.82) Multiple Sequence Alignments - Introns Inserted


Sequence 1: NCU02232.1	576 aa
Sequence 2: MG00881.1	507 aa
Sequence 3: FG06339.1	498 aa
Sequence 4: AN3033.1	521 aa
Alignment Length: 590 aa
Number Identitical Residues: 244 aa
Alignment Score (without introns) 12222


MG00881.1 	MPHSISPEGSQTG----AGAD---AAAAARPAPPTSNDTKQSPGEDIVMEDTEAVPSK--
NCU02232.1	MPHSESPPESENSPIPMANVDQEQEDAVAESTTAQVPEAQQPADEDVDMADSNAVLSAPT
FG06339.1 	MPHSIEAEAMSSP----SNVK---DEPVEETG--ISDNTPNATEVDTKMSDSEDVKKE--
AN3033.1  	MPHWVSSDGSSTS----------HNDVEALPDAPPLTDVSNGAEKDVAAAGKT-------
          	***  ..   ..                  . ..   :. : .  *    ..        

MG00881.1 	---------QEESQSEE-------------------QIKKEVKLDDLFADDDSDE-EFPS
NCU02232.1	NGTTHISESKAESQAETPNNQDTPVPEAATGAPSESKPATGVKLEELFDEMDSDDDEFPT
FG06339.1 	----------------------------------------AKQLEDLFD--DSDD-EFPT
AN3033.1  	--------------------------------------TAGVKIEDIFDDDEDEETEFPA
          	                                          :::::* . :.:: ***:

MG00881.1 	SRPVEDSKPSSPPAAPS~SSF2N~LPSTLHAS-DPEVMRSFYQRLFPWRYLFQWLNHSPN
NCU02232.1	TKAAK-REPASSPDLLS~SQS2D~MEIDLHEASDPEVMRTFYQRLFPWRYLFQWLNHSPT
FG06339.1 	SSAAI--QPSSPVAPLF2DAM~S~LKAS-----DPEVMRSFYQRLFPWRYLFQWLNHSPT
AN3033.1  	S--------SAPAETRV~GSA~E2ASAPVPVQVDTETMLQFYQRLFPFRYLFQWLNHGIV
          	:        ::.      .   .          *.*.*  *******:*********.  

MG00881.1 	PTNDFAHREFAFTLQNDAYLRYQSFTTSDL2LRKDVLRLMPSRFEIGPVYNTNPRERKTL
NCU02232.1	PTNDFGHREFAFTLQNDAYLRYQSFTTSDL2LRKDVLRLMPSRFEIGPVYTANPRDRKTL
FG06339.1 	PTNDFGNREFAFTLQNDAYLRYQSFPTADT2LRKDVLRLMPSRFEIGPVYTTNPRDRKTL
AN3033.1  	PSPDFGNREFALTLQNDAYLRYQSYPTADL2FRKDILKMNPSRFEIGPVYNRNPRDRKTL
          	*: **.:****:************:.*:*  :***:*:: **********. ***:****

MG00881.1 	RNSSAFKPLAKELCFDIDLTDYDDIRTCCDKANICNKCWQFMTMAIKTVDVALRDDFGFK
NCU02232.1	RNSSAFRPLSKELCFDIDLTDYDDIRTCCDKANICQKCWQFITMAIKVVDTALREDFGFK
FG06339.1 	RNSSAFKPLAKELCFDIDLTDYDDIRTCCDKANICNKCWQFMTMAIKVVDVALREDFGFK
AN3033.1  	G-SGQLKPLAKELVFDIDLTDYDDIRTCCTKANICAKCWAFVTMAIKVVDTALRQDFGFQ
          	  *. ::**:*** *************** ***** *** *:*****.**.***:****:

MG00881.1 	HIMWVYSGRRGAHAWVCDKKARVMDDQKRRAIAGYLEVVRGGAQSGKKVNVRRPLHPHLS
NCU02232.1	HIMWVYSGRRGAHAWVCDKSARALDDQKRRAIAGYLEVIRGGAQSGKKVNLRRPLHPHLV
FG06339.1 	HIMWVYSGRRGAHAWVCDKKVRSMDDQKRRAIAGYLEVVKGGAQSGKKVNVRRPLHPHLA
AN3033.1  	HILWVYSGRRGAHAWVCDSRARNLSDDRRRGIAGYLDLVRGGTNSGKRVNLKRPLHPHMT
          	**:***************. .* :.*::**.*****::::**::***:**::******: 

MG00881.1 	2RSLDVLKGHFQEDVLRAQDPWESEEQAEKLLQLLP--DRTLNDSLRKKWASSPGRSSLS
NCU02232.1	~RSLDILKQHFQSDVLEAQDPWRTEDQQEKLLALLPSDQKQLVSALRNKWASSPDRPSTL
FG06339.1 	2RSLEILKTHFQEDVLEVQDPWASPEQSEKLLQLLP--NQNLNESLRRKWDAAPGRPSTS
AN3033.1  	~RSLEILKPYFVQTTLVDQDTFASPEQEQRLLSLLP--DKGLNDSLRRKWESAPDRSSTN
          	 ***::** :* . .*  **.: : :* ::** ***  :: * .:**.** ::*.*.*  

MG00881.1 	KWADIDTVAKTGASRNLDAKALLEAKQDIVLEYTYPRLDIEVSKKLNHLLKSPFVVHPGT
NCU02232.1	KWADIDSVAKTSTAKNFDTKSLLDAKQDIVLEYTYPRLDIEVSKKLNHLLKSPFVVHPGT
FG06339.1 	KWADIDAVAKSGASKNLDARQLLEAKQDIVLEYTYPRLDIEVSKKLNHLLKSPFVVHPGT
AN3033.1  	KWADIDALAKAGKSSTLNPATLREAKQDIVLEYTYPRLDSEVSKKMIHLLKSPFVIHPGT
          	******::**:. : .::.  * :*************** *****: ********:****

MG00881.1 	G-----------------------R0ELLEEIDAWKQPEVKKEQGDDAAAQ---------
NCU02232.1	GRVCVPIDVKRGLESFDPLGVPTVQ~SLIREIDEWKKPEPK-EGRQEQEVE---------
FG06339.1 	GRVCVPINTKN-LEDFDPLGVPTVQ~GLLAEIDSWKS--------EGEEGG---------
AN3033.1  	GRVCVPIDIRN-VEKFDPLSVPTVS~QLLSEIDSWDSDHPSSGAAETAEGEGSAPNASDA
          	*  . . .     .. .. . .:    *: *** *..   ..   :     .::..::.:

MG00881.1 	---RPMQDWEKTSLKPHIDQFRVFVSALMKDERDPKIKREREE-------AESMEF
NCU02232.1	---KHVVDWEKTSLKGYIEFFRSFVIGLMKDEREVKVKREREEEGGGGGGGESMEF
FG06339.1 	---KGIADWEKTSLRPYIEQFRHFVNGLMKDEKDIKVKRERDE--------ESMEF
AN3033.1  	GGTRKLQDYEKTSLKPYIDYFRSFIAGLNKEERNG--KRERHEDSTPGVKSESMDF
          	..:: : *:*****: :*: ** *: .* *:*::   ****.*..  .  .***:*
```
